# Trap Depth Distribution Determines Afterglow Kinetics: A Local Model Applied to ZnGa_2_O_4_:Cr^3+^

**DOI:** 10.1021/acs.jpclett.4c01296

**Published:** 2024-08-29

**Authors:** Manuel Romero, Victor Castaing, Gabriel Lozano, Hernán Míguez

**Affiliations:** Institute of Materials Science of Seville, Spanish National Research Council−University of Seville, C. Américo Vespucio 49, 41092 Seville, Spain

## Abstract

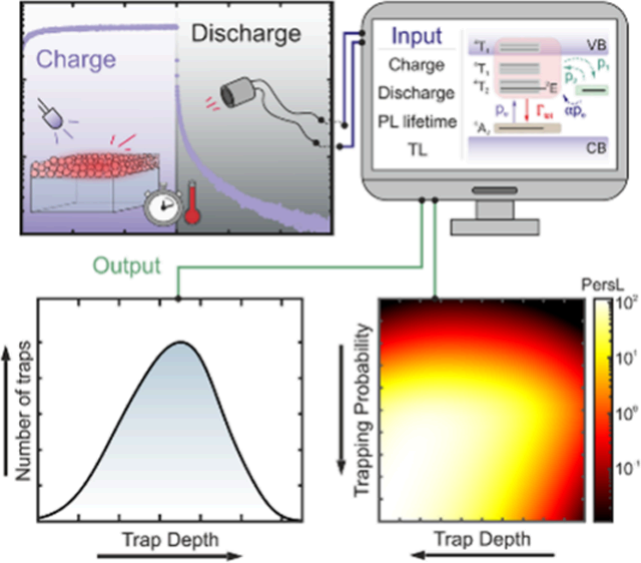

Persistent luminescence materials have applications in diverse fields such as smart signaling, anticounterfeiting, and in vivo imaging. However, the lack of a thorough understanding of the precise mechanisms that govern persistent luminescence makes it difficult to develop ways to optimize it. Here we present an accurate model to describe the various processes that determine persistent luminescence in ZnGa_2_O_4_:Cr^3+^, a workhorse material in the field. A set of rate equations has been solved, and a global fit to both charge/discharge and thermoluminescence measurements has been performed. Our results establish a direct link between trap depth distribution and afterglow kinetics and shed light on the main challenges associated with persistent luminescence in ZnGa_2_O_4_:Cr^3+^ nanoparticles, identifying low trapping probability and optical detrapping as the main factors limiting the performance of ZnGa_2_O_4_:Cr^3+^, with a large margin for improvement. Our results highlight the importance of accurate modeling for the design of future afterglow materials and devices.

Persistent luminescence (PersL) materials are unusual light emitters that have the ability to continue luminescing long after photoexcitation has ceased, behaving like light batteries. They are often used for night signaling and decoration and promise new opportunities in nanomedicine, security, or data storage.^1−6^ In general, PersL occurs when a photoluminescent center (e.g., Eu^2+^ or Cr^3+^ ions in an inorganic matrix) coexists with structural defects that act as electron or hole traps, storing these charges and thermally releasing them after a period of time. Canonical examples of such traps are Dy^3+^ ions in SrAl_2_O_4_:Eu^2+^,Dy^3+^ (SAO:Eu,Dy)^7^ and antisite defect pairs in ZnGa_2_O_4_:Cr^3+^ (ZGO:Cr).^8^

Since the pioneering studies of PersL by Randall and Wilkins in 1945,^9,10^ considerable efforts have been made to understand and model the mechanisms governing afterglow.^11−15^ According to the one trap–one recombination center model, the rate of charge detrapping, i.e., the process by which a charge is released from a trap, at a temperature *T* is typically assumed to be given by the following expression, which is known as *nth-order kinetics*:^14^
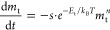
1where *s* is the frequency factor, *k*_B_ is the Boltzmann constant, *E*_t_ is the trap depth, *n* is the kinetic order, and *m*_t_ is the density of charged traps. First-order kinetics (*n* = 1) arises from the assumption that the probability of recombination is significantly greater than the probability of charge retrapping, i.e., the process by which a stimulated charge is trapped again, which is considered negligible. When retrapping dominates, second-order kinetics (*n* = 2) applies. Khanin et al. used second-order kinetics to demonstrate the effect of trap depth in PersL garnets and to correlate the trap depth distribution with afterglow dynamics.^16,17^ Finally, in intermediate cases where retrapping cannot be ignored, general order kinetics is used. Although detrapping experiments are often rationalized using this simple model,^9−14,16,17^ only first- and second-order kinetics have real physical meaning, which poses a challenge.

The main limitation of kinetic models is that they ignore charge dynamics,^18^ and they do not distinguish between where charges are trapped (charge density function) and where they could be trapped (trap depth distribution).^19^ These processes can be accounted for using ground-state and excited- state rate equations. For example, Tydtgat et al. described the charge dynamics in Sr_2_MgSi_2_O_7_:Eu^2+^,Dy^3+^ by solving rate equations based on first-order kinetics, introducing for the first time optically stimulated detrapping (OSD) and considering a single trap state.^20^ Notice that when a trap interacts only with its nearest recombination center, as happens when Eu^2+^ ions are directly excited in Sr_2_MgSi_2_O_7_:Eu^2+^,Dy^3+^, the PersL mechanism is called local, whereas in a nonlocal PersL mechanism the interaction between distant traps and recombination centers is allowed. Note that local trapping–detrapping typically yields first-order kinetics.^21^ Although these examples highlight the relevance of modeling, a general method for rationalizing PersL properties remains elusive. In this context, while the origin of PersL in ZGO:Cr, the most widely used material for in vivo imaging,^22,23^ has been studied,^8,24,25^ a mathematical model has never been applied to describe its afterglow dynamics.

In this work, a local model based on rate equations that consider a distribution of trap depths and the various processes that determine afterglow emission, including charge dynamics, retrapping, and OSD, is applied to ZGO:Cr. A single set of parameters is found to simultaneously reproduce the thermoluminescence (TL) and PersL kinetics, allowing the identification of the processes limiting the performance of the material. Rigorous modeling provides unique insight into the magnitudes that determine PersL performance and points to experimental pathways to improve afterglow. Our results lay the groundwork for the development of accurate models that will allow a deeper understanding of the mechanisms governing the afterglow, enabling novel PersL materials with improved performance.

The PersL mechanism in ZGO:Cr has been attributed to Cr^3+^ ions near antisite defect pairs.^8,25^ Unlike Eu^2+^ ions in SAO:Eu,Dy, where charge trapping occurs via photooxidation of Eu in favor of the photoreduction of Dy ions, Cr^3+^ does not change its oxidation state during PersL in ZGO, as the process involves both electrons and holes. When a Cr^3+^ ion near an antisite defect pair is excited by a photon, there is a certain probability that the following process will occur:
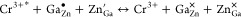
2where Cr^3+*^ is an excited Cr^3+^, Ga_Zn_^•^ is a Ga^3+^ in a Zn^2+^ site, and Zn_Ga_^′^ is a Zn^2+^ in a Ga^3+^ site, according to the Kröger–Vink notation. Electron migration from Zn_Ga_^′^ to Cr^3+^ during trapping causes Cr^3+^ to retain its original oxidation state. As a result, the emitting ions remain optically active during the process (see the Supporting Information).^8^Figure 1a shows a schematic of the energy levels of Cr^3+^ and the valence band (VB) and the conduction band (CB) of the ZGO host matrix. Although both electrons and holes are involved in the PersL process,^25^ only electron trapping is depicted in the sketch for simplicity. When excited with an energy below the band gap (∼4.5–5 eV),^26−28^ Cr^3+^ electrons from the ^4^A_2_ level are promoted to the ^4^T_1_(^4^P) (∼3.8 eV), ^4^T_1_(^4^F) (∼2.95 eV), and ^4^T_2_(^4^F) (∼2.21 eV) bands at a rate *p*_e_. However, most of the de-excitation of these electrons will take place mainly through the ^2^E level with a rate Γ_tot_, emitting photons in the process. While ^4^T_2_ → ^4^A_2_ photon emission is also possible, only ^2^E → ^4^A_2_ is considered in our model because it is more likely to be the only band observed in the afterglow regime. In addition, trap charging from the excited levels can occur at rate *p*_1_, and similarly phonon-assisted detrapping occurs at rate *p*_2_, while optical detrapping has an associated rate α*p*_e_ as it is assumed to be proportional to the excitation rate,^20^ with α being the ratio between the absorption cross section of the charged traps and that of the Cr^3+^ ions. Taking all the above into account, our model considers three energy levels for each Cr^3+^ ion: a ground state (represented by the ^4^A_2_ level), an excited state (consisted of the ^4^T_1_, ^4^T_2_, and ^2^E levels), and a single trapped state. Finally, it is important to note that although the ^4^T_1_ band lies within the CB of the host matrix, the necessity of hole migration from Cr^3+^ to Zn_Ga_^′^ constrains the PersL process to being purely local, since no possible nonlocal hole migration could take place through the VB when the material is excited below the band gap.

**Figure 1:**
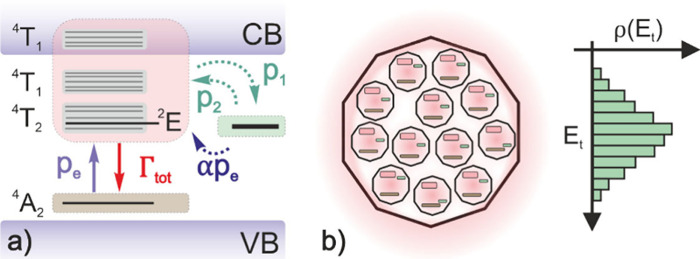
(a) Schematic of the energy levels involved in the of ZnGa_2_O_4_:Cr^3+^. (b) Schematic of a nanoparticle consisting of distribution of subsystems with different trap depths. Brown areas represent the ground state considered in the model, while red and green areas represent excited and trapped states, respectively.

Based on these considerations, the rate of change of the density *m*_e_ of excited Cr^3+^ ions and the density *m*_t_ of charged traps, assuming a single trap state, are given by
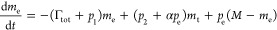

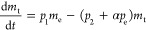
3where *M* is the total density of Cr^3+^ ions. Densities represent charges per unit volume. Thus, they have units of length to the power of −3. However, since the rest of the parameters of our model are volume-independent, we keep the densities unitless, as it only introduces a scaling factor. The rates *p*_1_ and *p*_2_ are expressed by
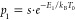

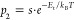
4and *T*_0_ = 295 K is a fixed parameter. This choice was made in order to have comparable parameters (*E*_1_ and *E*_t_) while keeping *p*_1_ temperature-independent, since the TL intensity charging at low and room temperature was comparable (see the Supporting Information).

The assumption of single trap depth is closer to ideal single crystal materials than to nanomaterials. For this reason, in our model we consider that each ZGO nanoparticle consists of a number of Cr^3+^ ions in different trap environments, which are associated with particular crystal distortions. Consequently, we assume that our material is composed of many non-interacting subsystems with a certain distribution ρ(*E*_t_) of trap depths (Figure 1b). In particular, we propose a model of *N* three-level systems, each of them with a trap depth between *E*_t_^*i*^ and *E*_t_^*i*^ + Δ*E* with a density of Cr^3+^ ions (*M*^*i*^) given by the product ρ(*E*_t_^*i*^)·*M*, where *M* is the total density of Cr^3+^ ions. Thus, the rate equations associated with each subsystem are
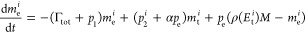

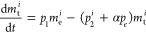
5where the superscript *i* refers to the *i*-th subsystem and *i* = 1···*N*.

We prepared ZGO:Cr nanoparticle films (see Materials and Methods) on fused silica substrates and performed an in-depth optical characterization to validate our model. Figure 2a shows the luminescence (Lum) spectra, normalized to the R line (691.5 nm), under 330 and 420 nm continuous excitation at 80 K. Note that Lum refers to the light emitted by the sample during photoexcitation and includes not only photoluminescence (PL) but also PersL and light emitted due to OSD, also known as optically stimulated luminescence (OSL). We chose Cr^3+^ excitation bands below the band gap of the host matrix to avoid the generation of free electrons and holes in the host^8,24,25,29^ so that the model remains purely local. The maximum is located at 695 nm, which corresponds to the N2 line associated with Cr^3+^ ions near antisite defect pairs. The Stokes phonon side bands (S-PSBs) are observed at wavelengths longer than 697 nm, while the anti-Stokes phonon side bands (AS-PSBs) appear at wavelengths shorter than 690 nm, as expected. Also, under 330 nm excitation the intensity of the N2 line increases compared to that of the R line, suggesting that the absorption of Cr^3+^ near antisite defect pairs is more efficient at this particular wavelength.^24^ It is noteworthy that the Lum spectrum also exhibits a broad band centered at ∼630 nm, which is absent in the PersL spectrum measured at room temperature (see Figure 2b). Although the origin of this band is debatable and further research is needed to unravel it, it does not originate from the Cr^3+^^2^E → ^4^A_2_ transitions, which are inherently narrow. Therefore, it is outside the scope of our model and does not affect the results of our analysis. To determine the Γ_tot_ of the excited state of Cr^3+^, we monitor the time-dependent Lum at 695 nm (see Figure 2c) for two different photoexcitation wavelengths, i.e., 330 and 420 nm. The measurements were performed at 80 K to minimize the contribution of the PersL. We use a biexponential model to obtain the decay rate of the Cr^3+^^2^E → ^4^A_2_ transitions involved in the afterglow (see Methods). In fact, the two exponentials account for Cr^3+^ cations in slightly different crystalline environments, which provides a good fit to the time-dependent intensity measurements for 420 nm light excitation (blue line in Figure 2c). For this photoexcitation condition, the PL and PersL spectra show only bands associated with Cr^3+^^2^E → ^4^A_2_ transitions. However, to fit the experimental data under 330 nm light excitation (purple line in Figure 2c), we must add an additional exponential to account for the fast contribution associated with the broad band centered at 630 nm that appears in the PL spectrum when it is measured at low temperature under UV excitation. We obtain a similar Γ_tot_ (∼200 Hz) regardless of the photoexcitation wavelength when the short component associated with the broad emission is excluded from the analysis. Notice that the relative weight of each component of the decay (Γ_1_/Γ_2_) is comparable and close to 50% (41%/59% vs 52%/48%). The results of the fits are given in Table 1. The lifetime associated with the 691.5 nm transition is very similar to that at 695 nm (N2 line). It is very likely that the crystalline quality of our nanoparticles prevents us from appreciating the dependence of the decay rate expected for sites with different lattice symmetries and thus we do not consider it in our model.

**Figure 2:**
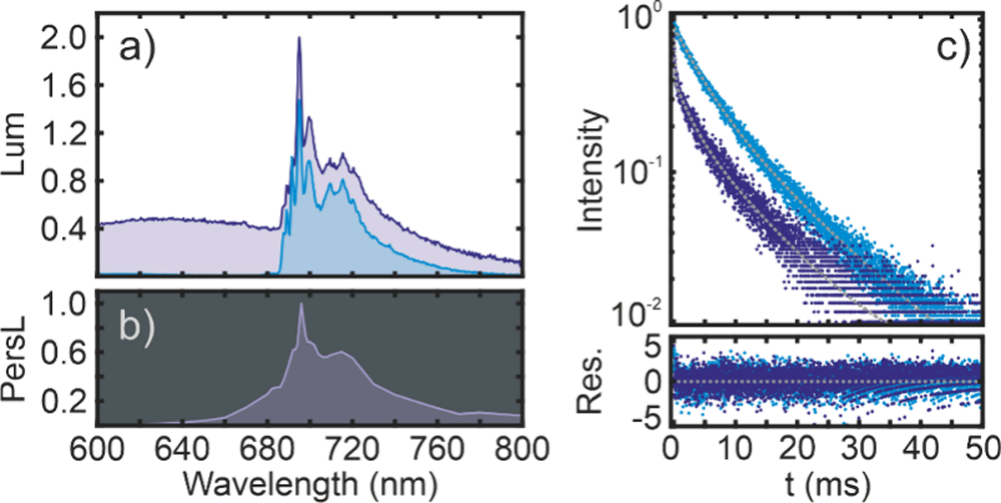
(a) Luminescence spectra normalized to the R1 line (691.5 nm) measured at 80 K. (b) Persistent luminescence spectrum normalized to the maximum. (c) Time-dependent luminescence measured at 80 K and 695 nm. Purple curves correspond to measurements under 330 nm excitation, while blue curves correspond to measurements under 450 nm.

**Table 1:** Exponential Fit Results from Measurements in Figure 2c

exc. wav. (nm)	Γ_1_(Hz)/w_1_ (%)	Γ_2_(Hz)/w_2_ (%)	Γ_3_(Hz)/w_3_ (%)
450	88.6/40.8	248.1/59.2	
330	104.2/24.8	382.0/23.0	7534/52.1

To prove the consistency of our model, we performed a simultaneous fit of both charge/discharge and TL measurements of our samples under different excitation conditions. Figure 3a shows the kinetics of our material for a photoexcitation wavelength of 330 nm and different excitation intensities to study the effect of OSD. In addition, TL measurements displayed in Figure 3b were performed for two charging temperatures (low and room temperature) to obtain information about shallow traps that contribute to the initial stages of the afterglow. The calculation of the parameters included in eq 5 was done by least-squares minimization (see Table 2). Γ_tot_ was also treated as a fitting parameter, but with certain constraints. Analytical solutions of eq 5 were used to reproduce the charge/discharge curves (see the Supporting Information), while numerical integration of the equations was performed to calculate the TL curves (see Materials and Methods). Figure 3 shows the results of the fits. The good agreement between experiments and calculations validates our model and confirms that PersL in ZGO:Cr is entirely local for photoexcitation wavelengths below the band gap. Nevertheless, we observe a certain discrepancy between measurements and calculations for long time values of the PersL decay, since parameters such as α and *E*_1_ are not considered to be trap-depth-dependent. In any case, it is noteworthy that the same set of parameters is used to reproduce both charge/discharge and TL curves without any further assumptions than the proposed local model for the PersL mechanism.

**Figure 3:**
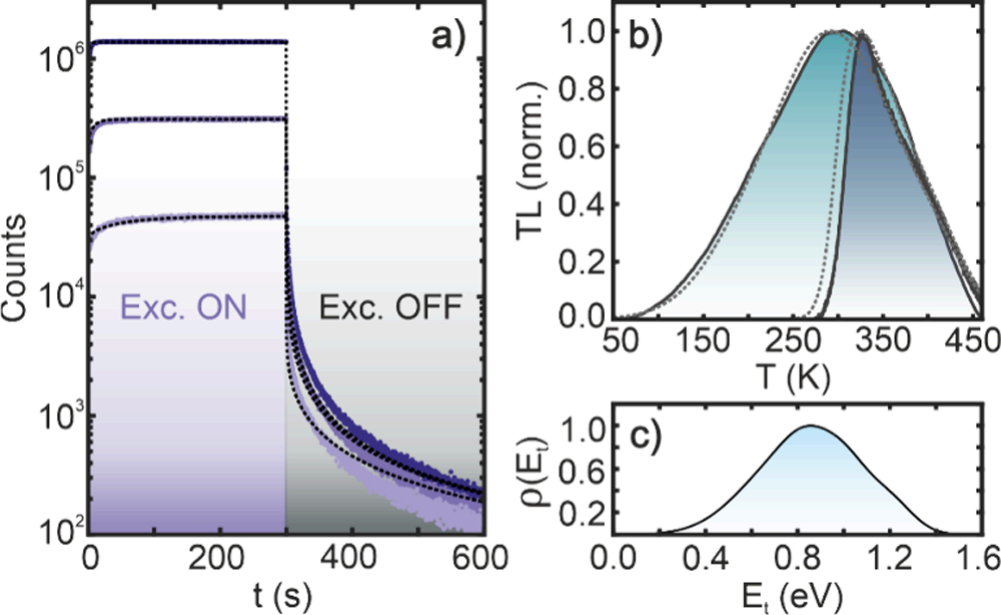
(a) Charge/discharge curves for three different excitation intensities (darker curves correspond to higher excitation intensities). (b) Thermoluminescence (TL) curves for two charging temperatures (light blue corresponds to charging at 15 K, while dark blue corresponds to charging at 295 K and then cooling to 245 K before heating). (c) Normalized trap depth distribution (ρ) obtained from the fits. The corresponding fit results are shown as dotted lines in (a) and (b).

**Table 2:** Fitting Parameters Used to Calculate the Theoretical Curves Shown in Figure 3

*p*_e_ (max.) (Hz)	Γ_tot_ (Hz)	*s* (Hz)	*M*	α	E_**1**_(eV)
3.02 × 10^–2^	248.3	10^13^	10^10^	30	0.62

The simultaneous fitting of both experiments ensures that the calculated distribution (see Figure 3c) corresponds to the actual trap depth distribution and not to the electron population function.^19^ Note that our analysis includes TL measurements in order to infer information about the full range of trap depths. The preparation and processing conditions of the ZGO:Cr nanomaterial are responsible for the wide trap depth distribution. Indeed, the synthesis conditions of ZGO:Cr nanoparticles could lead to a lower crystallinity and a higher number of defects compared to bulk materials. Although only antisite defect pairs are responsible for PersL, a more defective lattice could favor the appearance of a large variety of traps. As a consequence, the trap depth distribution fully determines not only the TL, where such a distribution induces a clear deviation from the asymmetric bell shape typically associated with first-order kinetics, but also the PersL dynamics, which have a clear multiexponential character (see Figure 3a). To shed more light on this, we performed a detailed analysis of the trap dynamics as a function of time and trap depth. Figure 4a shows the calculated normalized trap densities *m*_t_^*i*^ as a function of the time after the excitation ceased. Our model confirms that traps with energies below ∼0.6 eV cannot remain charged at room temperature, since their detrapping rate is too fast to contribute to the afterglow. In addition, a significant number of traps with energies above ∼1.2 eV have high charge densities that are unlikely to be released in less than a year (∼10^7^ s). In fact, our model shows that the trap depth range that actually contributes to PersL on a reasonable time scale (from 0 to ∼10^5^ s) is only between 0.7 and 1.0 eV, which is a small fraction of the total trap depth distribution. This striking fact is an indication of the potential for optimization of the material. To provide further insight, Figure 4b shows the calculated afterglow intensity (*I*_PersL_) as a function of the trap energy, since the number of photons emitted is proportional to the density of electrons in the excited state.
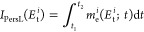
6The calculations in Figure 4b were performed for *t*_1_ = 0, 1, 30, 60, and 300 s,and *t*_2_ = 1000 s. Our results confirm that most of the PersL is emitted in the first minute after excitation ceases, coming from the traps with energies between 0.7 and 0.8 eV, while slower and weaker afterglow comes from traps with energies between 0.8 and 1.0 eV (see the Supporting Information). Similarly, our method can be extended to other PersL materials such as SAO:Eu,Dy, which is typically modeled by assuming a single trap state and a multiexponential PersL decay.

**Figure 4:**
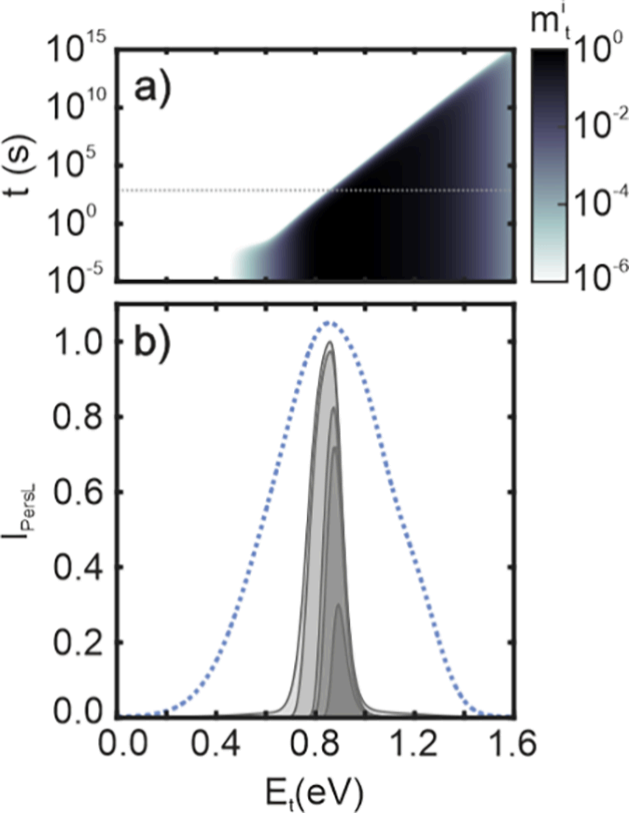
(a) Normalized charged trap density (*m*_t_^i^) as a function of the energy of the traps (*E*_t_) and time after excitation stops (*t*). Calculations consider that the system is excited for 300 s. The dotted gray line highlights *t* = 300 s after excitation stops. Before normalization, the actual values are on the order of the total density of , i.e., *M* = 10^10^. (b) Integrated persistent luminescence calculated for different times after excitation. From lighter to darker gray: 0 s, 1 s, 30 s, 1 min, and 5 min. The full trap depth distribution is also plotted (dotted blue line) for visual reference.

With the goal of finding new ways to optimize PersL in ZGO:Cr, we quantify the effect of shifting the *E*_1_ and *E*_t_ energies on the afterglow intensity (*t*_1_ = 1 s and *t*_2_ = 3000 s). It is well-known that it is possible to modify the trap depth through host matrix engineering^30,31^ or doping engineering.^32−34^ Also, it is generally accepted that increasing the trapping probability, equivalent to varying *E*_1_, results in improved PersL. However, it is not straightforward to change this parameter independently without also affecting the trap depth. Figure 5 shows the computed *I*_PersL_ values for different trap distributions. In particular, we plot *I*_PersL_ as a function of the shifts Δ*E*_t_ and Δ*E*_1_ with respect to the original trap depth and trapping probability. Our results indicate that the afterglow increases significantly with the trapping probability, i.e., for lower values of *E*_1_. In fact, a reduction of *E*_1_ by 0.2 eV results in an increase of PersL by almost two orders of magnitude, suggesting large room for improvement. It is also noticeable that the maximum of the PersL enhancement shifts to lower values of the trap depth when *E*_1_ is reduced, which is caused by retrapping. As the probability of trapping increases, so does the probability of retrapping. For this reason, if *E*_1_ is strongly reduced, retrapping would dominate PersL and radiative recombination would be inhibited. Therefore, increasing the detrapping probability (reducing the trap depth) is necessary to counteract retrapping. Nevertheless, *E*_1_ is found to be the main parameter affecting PersL intensity. Taking all this into account, although modifying the trap depth is experimentally more accessible in most cases, favoring trapping probability is generally more effective to enhance PersL.

**Figure 5:**
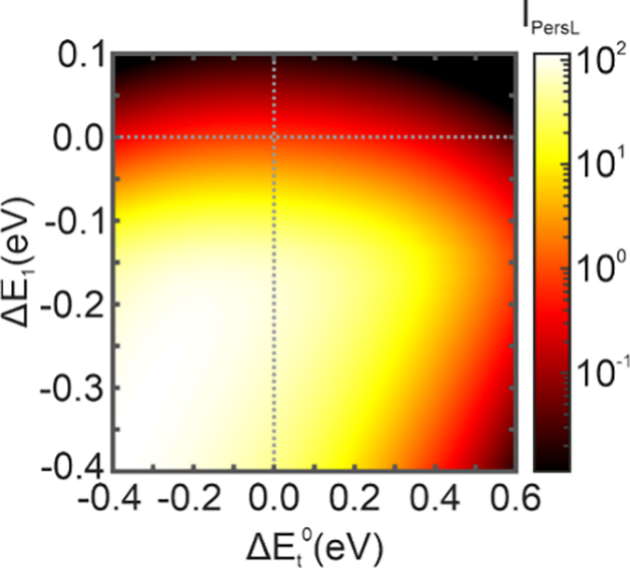
Computed *I*_PersL_ values for different trap distributions. Δ*E*_t_ and Δ*E*_1_ represent energy shifts with respect to the original trap depth and trap probability, respectively.

Finally, we use our model to study the effect of optical detrapping. Although OSL has demonstrated great potential for theranostics,^35^ an analysis of the stringent effect of OSD on the trapping capabilities of PersLNPs remains elusive. Our results indicate that optical detrapping affects not only trap release but also charging. In particular, Figure S4 shows time-dependent calculations of light emission assuming the fitting parameters given in Table 2, but with α = 0 to suppress the OSD effect. Indeed, in the absence of OSD, the charging curves show the same curvature regardless of the excitation power. In contrast, OSD induces a variation in the curvature of the charging curve (*t* < 300 s in our case) for different excitation intensities (see Figure S4). In addition, OSD causes charge detrapping to be faster under excitation, which simultaneously reduces the number of traps available under continuous excitation. Thus, OSD imposes a lower bound on the charge capacity and reduces the effect of varying the excitation intensity. Note that the PersL intensity at *t* ≈ 500 s is very similar despite the large differences in excitation power during charging, as shown in Figure 3a. In fact, simulations with α = 0 (dotted lines) show much higher afterglow than our measurements, with a strong dependence on the excitation power. Although solving this problem is a real challenge for the field, since OSD and photoexcitation are intertwined, our model highlights the importance of optimizing the charging conditions to mitigate its effects.

In conclusion, we have proposed an accurate model to describe the various processes that determine the PersL of ZGO:Cr, a workhorse material in the field, when it is photoexcited below the band gap of the host. A set of rate equations has been solved, and a global fit to both charge/discharge and TL measurements under different conditions has been performed. Our results establish a direct link between trap depth distribution and afterglow kinetics and shed light on the main challenges associated with PersL in ZGO:Cr nanoparticles: low trapping probability combined with a significant OSD. Furthermore, we have demonstrated that increasing the trapping probability is more effective to improve the PersL intensity than modifying the trap depth distribution. Our approach represents a general way to study PersL mechanisms based on the analysis of experimental data by solving rate equations derived from physical models. This opens the door to the optimization of PersL based on the combined efforts of precise chemical and material engineering and deep knowledge of the processes governing the afterglow.

## Materials and Methods

### Preparation of ZnGa_2_O_4_:Cr^3+^ Nanoparticle-Based Films

ZGO:Cr nanoparticles were prepared through a microwave-assisted hydrothermal route described elsewhere.^36^ Briefly, zinc acetate (0.6 mmol, Sigma-Aldrich, 99.99%), gallium nitrate (1.14 mmol, Sigma-Aldrich, 99.9%), and chromium nitrate (6.32·10^–2^ mmol, Sigma-Aldrich, 99%) were dissolved in 30 mL of Milli-Q water using magnetic stirring for 5 min. The resulting concoction was mixed with a sodium citrate solution (30 mL, 3 mmol, Sigma-Aldrich, 99.5%) during 30 min. Tetraethylammonium hydroxide (Fluka) was then added dropwise until the solution reached pH = 8.80. The resulting solution was stirred 30 min before being transferred to a tightly closed Teflon reactor and placed in a microwave oven (Sineo MDS-8) to allow heating at 220 °C for 30 min with a 13 °C·min^–1^ heating ramp. The dispersion was finally washed three times with Milli-Q water and two more times with absolute ethanol (VWR Chemicals).

A dispersion containing a controlled mass of ZGO:Cr nanoparticles (*m*_Nps_) in 120 mL of absolute ethanol was mixed with a viscous solvent (α-terpineol, 3·*m*_Nps_, SAFC, ≥96%) and ethyl cellulose (0.45·*m*_Nps_, Sigma-Aldrich) using tip sonication to allow the preparation of a viscous paste through ethanol removal.^37^ The resulting paste was deposited on fused silica using the blade coating method and stabilized through sequential heating using a hot plate (80 °C for 1 h, 220 °C for 30 min, and 450 °C for 30 min with a 5 °C·min^–1^ heating ramp). Finally, the film was crystallized in a muffle furnace (900 °C for 12 min with a 5 °C·min^–1^ heating ramp, SNOL 8.2/1100).

### Luminescence Measurements

Charge/discharge curves were measured with an Edinburgh FLS1000 spectrofluorometer, and the temperature was controlled using a cryostat (Optistat-DM, Oxford Instruments) attached to the fluorometer. An OD1 filter was used for charge/discharge measurements while the shutter was open to avoid saturation. The data were later corrected to take this into account. All the samples were thermally emptied before measurements.

Time-dependent PL was measured using a multichannel scaling method and a pulsed xenon lamp at 10 Hz repetition rate. Time window was set to 100 ms with 8000 channels and a maximum count number of 5000. Fittings were obtained by using the tail fitting method starting in the fifth channel after the maximum (50 μs). A multiexponential fitting was used in all the cases, which was given by the following expression:
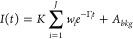
7where *I*(*t*) is the intensity in function of time, *K* is a scale parameter, *w*_*i*_ represents the weights, Γ_*i*_ represents the rates, *A*_bkg_ is the background signal, and *J* is the number of components. Note that PL decay rates are typically measured at low temperatures to avoid thermal detrapping, as it is necessary to find the measurement conditions that allow PL while preventing PersL. The afterglow signal increases the background of *I*(*t*), which typically hinders the determination of PL decay rates in persistent phosphors at room temperature. However, we have found that in our case it is also possible to measure the PL decay rate at room temperature using visible wavelengths, e.g., 420 or 560 nm, since the contribution of the PersL signal is negligible at such excitation wavelengths in this time scale.

### Thermoluminescence Measurements

A closed-cycle He-flow cryostat (Sumitomo Cryogenics HC-4E) and a temperature controller (Lakeshore 340) were used both to cool down and heat up samples in TL measurements. After being charged by a 312 nm UV lamp (Vilber Lourmat), TL glow curves were acquired by a charge coupled device camera (Roper Pixis 100) coupled with a monochromator (Acton Spectra Pro, Princeton Instruments).

### Calculations and Fittings

Charge/discharge measurements were reproduced using the analytical solutions to eq 5 (see the Supporting Information). TL measurements were fitted by finding numerical solutions to eq 5 assuming linear heating/cooling. To reduce the fitting parameters to obtain ρ(*E*_*t*_), the distribution was assumed to be the sum of four Gaussian distributions, each with three fitting parameters (height, variance, and mean). All of the results were calculated using in-house-developed MATLAB codes. The included *ode15s* function was used to find numerical solutions, while the *ga* (genetic algorithm) function was used to find the parameters that best fit the experimental measurements.

## Data Availability

The data supporting the results of this study are openly available in the Digital CSIC repository at https://doi.org/10.20350/digitalCSIC/16421.

## Supporting Information Available

Schematic of the trapping model, TL curves for charging at 15 and 295 K, analytical solution of the model equation, charging time constant as a function of the trap depth, results on the effect of OSD in charge/discharge curves, PL spectra, zoomed-in view of Figure 4b (PDF)
Transparent Peer Review report available (PDF)

## References

[ref1] Poelman D.; Van der Heggen D.; Du J.; Cosaert E.; Smet P. F. Persistent Phosphors for the Future: Fit for the Right Application. J. Appl. Phys. 2020, 128 (24), 240903 10.1063/5.0032972.

[ref2] Zhou Z.; Li Y.; Peng M. Near-Infrared Persistent Phosphors: Synthesis, Design, and Applications. Chemical Engineering Journal 2020, 399, 125688 10.1016/j.cej.2020.125688.

[ref3] Huang K.; Le N.; Wang J. S.; Huang L.; Zeng L.; Xu W.; Li Z.; Li Y.; Han G. Designing Next Generation of Persistent Luminescence: Recent Advances in Uniform Persistent Luminescence Nanoparticles. Adv. Mater. 2022, 34 (14), 2107962 10.1002/adma.202107962. 34877721

[ref4] Zhuang Y.; Wang L.; Lv Y.; Zhou T.; Xie R. Optical Data Storage and Multicolor Emission Readout on Flexible Films Using Deep-Trap Persistent Luminescence Materials. Adv. Funct Materials 2018, 28 (8), 1705769 10.1002/adfm.201705769.

[ref5] Castaing V.; Arroyo E.; Becerro A. I.; Ocaña M.; Lozano G.; Míguez H. Persistent Luminescent Nanoparticles: Challenges and Opportunities for a Shimmering Future. J. Appl. Phys. 2021, 130 (8), 080902 10.1063/5.0053283.

[ref6] Castaing V.; Lozano G.; Míguez H. Transparent Phosphor Thin Films Based on Rare-Earth-Doped Garnets: Building Blocks for Versatile Persistent Luminescence Materials. Advanced Photonics Research 2022, 3 (7), 2100367 10.1002/adpr.202100367.

[ref7] Joos J. J.; Korthout K.; Amidani L.; Glatzel P.; Poelman D.; Smet P. F. Identification of Dy 3 + /Dy 2 + as Electron Trap in Persistent Phosphors. Phys. Rev. Lett. 2020, 125 (3), 033001 10.1103/PhysRevLett.125.033001. 32745429

[ref8] Gourier D.; Bessière A.; Sharma S. K.; Binet L.; Viana B.; Basavaraju N.; Priolkar K. R. Origin of the Visible Light Induced Persistent Luminescence of Cr3+-Doped Zinc Gallate. J. Phys. Chem. Solids 2014, 75 (7), 826–837. 10.1016/j.jpcs.2014.03.005.

[ref9] Randall J. T.; Wilkins M. H. F. Phosphorescence and Electron Traps - I. The Study of Trap Distributions. Proc. R. Soc. London A 1945, 184 (999), 365–389. 10.1098/rspa.1945.0024.

[ref10] Randall J. T.; Wilkins M. H. F. Phosphorescence and Electron Traps II. The Interpretation of Long-Period Phosphorescence. Proc. R. Soc. London A 1945, 184 (999), 390–407. 10.1098/rspa.1945.0025.

[ref11] Hornyak W. F.; Chen R. Thermoluminescence and Phosphorescence with a Continuous Distribution of Activation Energies. J. Lumin. 1989, 44 (1–2), 73–81. 10.1016/0022-2313(89)90023-9.

[ref12] Gobrecht H.; Hofmann D. Spectroscopy of Traps by Fractional Glow Technique. J. Phys. Chem. Solids 1966, 27 (3), 509–522. 10.1016/0022-3697(66)90194-6.

[ref13] Medlin W. L. Decay of Phosphorescence from a Distribution of Trapping Levels. Phys. Rev. 1961, 123 (2), 502–509. 10.1103/PhysRev.123.502.

[ref14] Chen R. Glow Curves with General Order Kinetics. J. Electrochem. Soc. 1969, 116 (9), 1254 10.1149/1.2412291.

[ref15] Garlick F. J.; Gibson F. The Electron Trap Mechanism of Luminescence in Sulphide and Silicate Phosphors. Proc. Phys. Soc. 1948, 60, 574 10.1088/0959-5309/60/6/308.

[ref16] Khanin V. M.; Vrubel I. I.; Polozkov R. G.; Shelykh I. A.; Venevtsev I. D.; Meijerink A.; Wieczorek H.; Boerekamp J.; Spoor S.; Rodnyi P. A.; Ronda C. Modeling and Assessment of Afterglow Decay Curves from Thermally Stimulated Luminescence of Complex Garnets. J. Phys. Chem. A 2019, 123 (9), 1894–1903. 10.1021/acs.jpca.8b11778. 30775917

[ref17] Khanin V.; Venevtsev I.; Spoor S.; Boerekamp J.; Van Dongen A.-M.; Wieczorek H.; Chernenko K.; Buettner D.; Ronda C.; Rodnyi P. A New Method for Unambiguous Determination of Trap Parameters from Afterglow and TSL Curves Connection: Example on Garnets. Opt. Mater. 2017, 72, 161–168. 10.1016/j.optmat.2017.05.040.

[ref18] Basun S.; Imbusch G. F.; Jia D. D.; Yen W. M. The Analysis of Thermoluminescence Glow Curves. J. Lumin. 2003, 104 (4), 283–294. 10.1016/S0022-2313(03)00082-6.

[ref19] Feng A.; Joos J. J.; Du J.; Smet P. F. Revealing Trap Depth Distributions in Persistent Phosphors with a Thermal Barrier for Charging. Phys. Rev. B 2022, 105 (20), 205101 10.1103/PhysRevB.105.205101.

[ref20] Tydtgat C.; Meert K. W.; Poelman D.; Smet P. F. Optically Stimulated Detrapping during Charging of Persistent Phosphors. Opt. Mater. Express 2016, 6 (3), 844 10.1364/OME.6.000844.

[ref21] Bos A. J. J. Theory of Thermoluminescence. Radiat. Meas. 2006, 41, S45–S56. 10.1016/j.radmeas.2007.01.003.

[ref22] Sharma S. K.; Gourier D.; Teston E.; Scherman D.; Richard C.; Viana B. Persistent Luminescence Induced by near Infra-Red Photostimulation in Chromium-Doped Zinc Gallate for in Vivo Optical Imaging. Opt. Mater. 2017, 63, 51–58. 10.1016/j.optmat.2016.06.053.

[ref23] Sun X.; Song L.; Liu N.; Shi J.; Zhang Y. Chromium-Doped Zinc Gallate Near-Infrared Persistent Luminescence Nanoparticles in Autofluorescence-Free Biosensing and Bioimaging: A Review. ACS Appl. Nano Mater. 2021, 4 (7), 6497–6514. 10.1021/acsanm.1c01115.

[ref24] Bessière A.; Jacquart S.; Priolkar K.; Lecointre A.; Viana B.; Gourier D. ZnGa_2__4_:Cr^3+^: A New Red Long-Lasting Phosphor with High Brightness. Opt. Express 2011, 19 (11), 10131 10.1364/OE.19.010131. 21643271

[ref25] Bessière A.; Sharma S. K.; Basavaraju N.; Priolkar K. R.; Binet L.; Viana B.; Bos A. J. J.; Maldiney T.; Richard C.; Scherman D.; Gourier D. Storage of Visible Light for Long-Lasting Phosphorescence in Chromium-Doped Zinc Gallate. Chem. Mater. 2014, 26 (3), 1365–1373. 10.1021/cm403050q.

[ref26] Omata T.; Ueda N.; Ueda K.; Kawazoe H. New Ultraviolet-Transport Electroconductive Oxide, ZnGa_2_O_4_ Spinel. Appl. Phys. Lett. 1994, 64 (9), 1077–1078. 10.1063/1.110937.

[ref27] Karazhanov S. Zh.; Ravindran P. Ab Initio Study Of Double Oxides ZnX_2_O_4_ (X = Al, Ga, In) Having Spinel Structure. J. Am. Ceram. Soc. 2010, 93 (10), 3335–3341. 10.1111/j.1551-2916.2010.03864.x.

[ref28] Zhang Y.; Wu Z.; Geng D.; Kang X.; Shang M.; Li X.; Lian H.; Cheng Z.; Lin J. Full Color Emission in ZnGa_2_O_4_: Simultaneous Control of the Spherical Morphology, Luminescent, and Electric Properties via Hydrothermal Approach. Adv. Funct Materials 2014, 24 (42), 6581–6593. 10.1002/adfm.201402092.

[ref29] Mikenda W.; Preisinger A. N-Lines in the Luminescence Spectra of Cr^3+^-Doped Spinels (I) Identification of N-Lines. J. Lumin. 1981, 26 (1–2), 53–66. 10.1016/0022-2313(81)90169-1.

[ref30] Ueda J.; Kuroishi K.; Tanabe S. Bright Persistent Ceramic Phosphors of Ce^3+^-Cr^3+^-Codoped Garnet Able to Store by Blue Light. Appl. Phys. Lett. 2014, 104 (10), 101904 10.1063/1.4868138.

[ref31] Pan Z.; Castaing V.; Yan L.; Zhang L.; Zhang C.; Shao K.; Zheng Y.; Duan C.; Liu J.; Richard C.; Viana B. Facilitating Low-Energy Activation in the Near-Infrared Persistent Luminescent Phosphor Zn_1+*x*_Ga_2–2*x*_Sn_*x*_O_4_:Cr^3+^*via* Crystal Field Strength Modulations. J. Phys. Chem. C 2020, 124 (15), 8347–8358. 10.1021/acs.jpcc.0c01951.

[ref32] Lecointre A.; Bessière A.; Bos A. J. J.; Dorenbos P.; Viana B.; Jacquart S. Designing a Red Persistent Luminescence Phosphor: The Example of YPO_4_:Pr ^3+^,Ln^3+^ (Ln = Nd, Er, Ho, Dy). J. Phys. Chem. C 2011, 115 (10), 4217–4227. 10.1021/jp108038v.

[ref33] Ueda J.; Hashimoto A.; Takemura S.; Ogasawara K.; Dorenbos P.; Tanabe S. Vacuum Referred Binding Energy of 3d Transition Metal Ions for Persistent and Photostimulated Luminescence Phosphors of Cerium-Doped Garnets. J. Lumin. 2017, 192, 371–375. 10.1016/j.jlumin.2017.07.006.

[ref34] Katayama Y.; Kayumi T.; Ueda J.; Tanabe S. Enhanced Persistent Red Luminescence in Mn^2+^-Doped (Mg,Zn)GeO_3_ by Electron Trap and Conduction Band Engineering. Opt. Mater. 2018, 79, 147–151. 10.1016/j.optmat.2018.03.033.

[ref35] Chuang Y.-J.; Zhen Z.; Zhang F.; Liu F.; Mishra J. P.; Tang W.; Chen H.; Huang X.; Wang L.; Chen X.; Xie J.; Pan Z. Photostimulable Near-Infrared Persistent Luminescent Nanoprobes for Ultrasensitive and Longitudinal Deep-Tissue Bio-Imaging. Theranostics 2014, 4 (11), 1112–1122. 10.7150/thno.9710. 25285164 PMC4173761

[ref36] Arroyo E.; Medrán B.; Castaing V.; Lozano G.; Ocaña M.; Becerro A. I. Persistent Luminescence of Transparent ZnGa_2_O_4_:Cr^3+^ Thin Films from Colloidal Nanoparticles of Tunable Size. J. Mater. Chem. C 2021, 9 (13), 4474–4485. 10.1039/D1TC00258A.

[ref37] Castaing V.; Romero M.; Torres J.; Lozano G.; Míguez H. Scattering Spheres Boost Afterglow: A Mie Glass Approach to Go Beyond the Limits Set by Persistent Phosphor Composition. Advanced Optical Materials 2024, 12, 2301565 10.1002/adom.202301565.

