# Supplementary material for: Trap Depth Distribution Determines Afterglow Kinetics: A Local Model Applied to ZnGa2O4:Cr3+
Source: J Phys Chem Lett. 2024 Aug 29;15(35):9129–35. doi: 10.1021/acs.jpclett.4c01296 (PMC11382276; doi:10.1021/acs.jpclett.4c01296)
Supplement: Supplementary file 1 — jz4c01296_si_001.pdf [file jz4c01296_si_001.pdf]

Supporting information of:

Trap depth distribution determines afterglow kinetics: a local model applied to $ZnGa_2O_4$: Cr^{3+}

Manuel Romero, Victor Castaing,* Gabriel Lozano,* and Hernán Míguez

Institute of Materials Science of Seville, Spanish National Research Council – University of Seville, C. Américo Vespucio 49, 41092, Seville, Spain

* victor.castain@icmse.csic.es; g.lozano@csic.es

Trapping model

When trapping occurs, an electron goes from the Cr^{3+} excited state to a $Ga^{\bullet}_{Zn}$ ion, but at the same time an electron goes from Zn'_{Ga} to the Cr^{3+} ground state. Finally, when detrapping takes place, the reverse process occurs. As a result, Cr^{3+} ions do not change their oxidation state during trapping and detrapping and remain optically active during the process (see Figure S1).

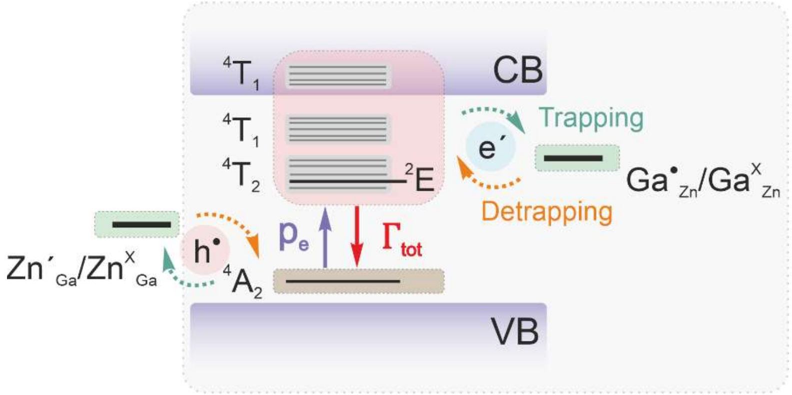

Figure S1. Schematic of the complete trapping model with Cr^{3+} ions near antisite defects. The shaded areas correspond to those in Figure 1 of the main manuscript.

Thermoluminescence measurements

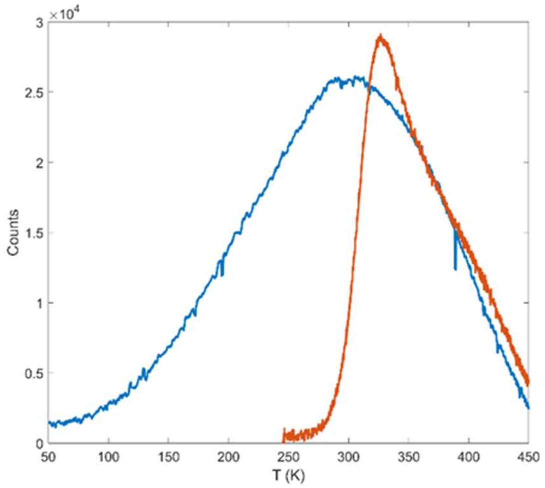

Figure S2. TL curves for two charging temperatures (blue line corresponds to charging at 15 K, while orange corresponds to charging at 295 K and then cooling down to 245 K before heating).

Analytical solution to the equations

A general solution to Equations 3 of the main manuscript is given in matrix form by:

$$\begin{pmatrix} m_e(t) \\ m_t(t) \end{pmatrix} = C_1 e^{\lambda_1 t} \boldsymbol{v_1} + C_2 e^{\lambda_2 t} \boldsymbol{v_2}$$

(S1)

where $\lambda_{1,2}$ and $\boldsymbol{v_{1,2}}$ correspond to the eigenvalues and eigenvectors, respectively, of the following matrix:

$$A = \begin{pmatrix} -(p_e + \Gamma_{tot} + p_1) & p_2 + \alpha p_e \\ p_1 & -(p_2 + \alpha p_e) \end{pmatrix}$$

(S2)

having the following expression:

$$\lambda_{1,2} = -\frac{1}{2}\left(p_1 + p_2 + (1+\alpha)p_e + \Gamma_{tot} \pm \sqrt{(p_1 + p_2 + (\alpha - 1)p_e - \Gamma_{tot})^2 + 4p_1\Gamma_{tot}}\right)$$

(S3)

The coefficients $C_{1,2}$ are determined by the initial conditions $m_e(t=0) = m_e^0$ and $m_t(t=0) = m_t^0$:

$$C_1 = \frac{m_e^0 - {}^{v_{2,x}}\!/_{v_{2,y}} \cdot m_t^0}{v_{1,x} - {}^{v_{2,x}}\!/_{v_{2,y}} \cdot v_{1,y}} + \frac{p_e M}{v\lambda_1} v_{2,y}$$

$$C_2 = \frac{m_e^0 - {}^{v_{1,x}}\!/_{v_{1,y}} \cdot m_t^0}{v_{2,x} - {}^{v_{1,x}}\!/_{v_{1,y}} \cdot v_{2,y}} + \frac{p_e M}{\nu \lambda_2} v_{1,y}$$

(S4)

where ν is the determinant of the matrix A.

Charging time as a function of trap depth

The time needed to reach steady state is determined by $\lambda_{1,2}$ according to Eq. S1. Although deep traps have a low detrapping rate, the charging time is limited by the other parameters (excitation rate, decay rate, etc.). In this way, traps with extremely long detrapping times (~years) reach steady state in reasonably short times (~hundreds of seconds), as can be seen in Fig. S3.

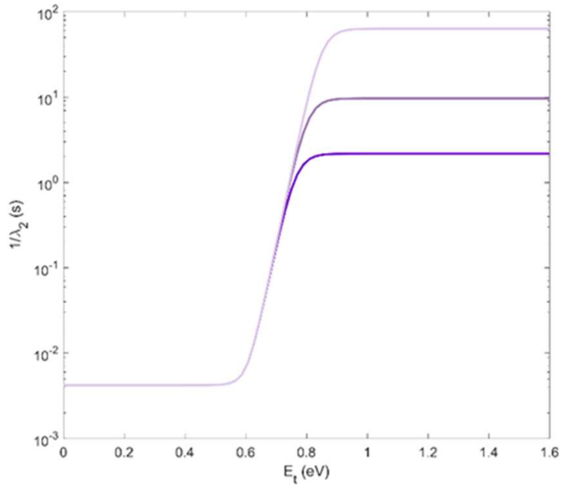

Figure S3. λ_2^{-1} as a function of trap depth E_t for the three values of p_e used in the main manuscript.

Normalized charging curves and optical detrapping effect

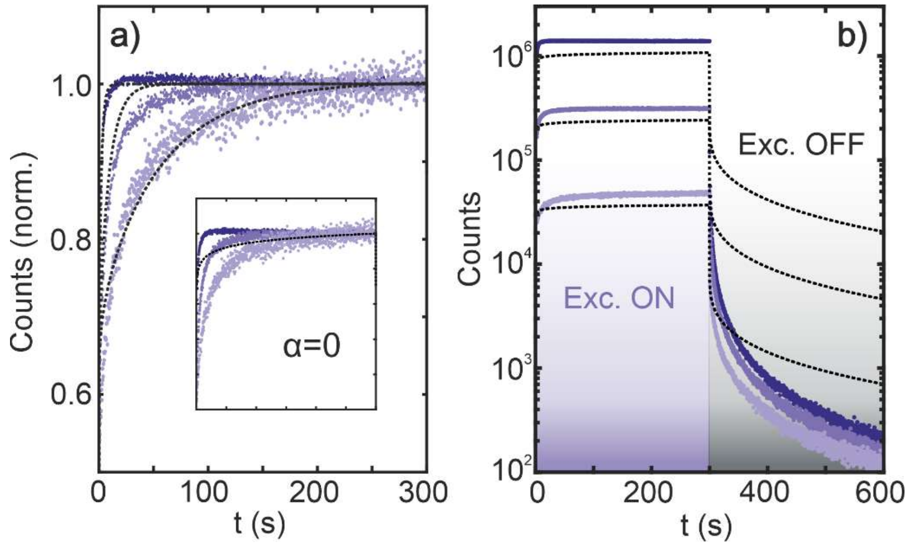

Figure S4. a) Normalized charging curves for different 330 nm light excitation intensities. Dotted lines correspond to fittings. Inset shows the effect of deactivating OSL in the model. b) Experimental charge/discharge curves shown in Figure 3a and simulations with α=0.

Luminescence excitation spectra

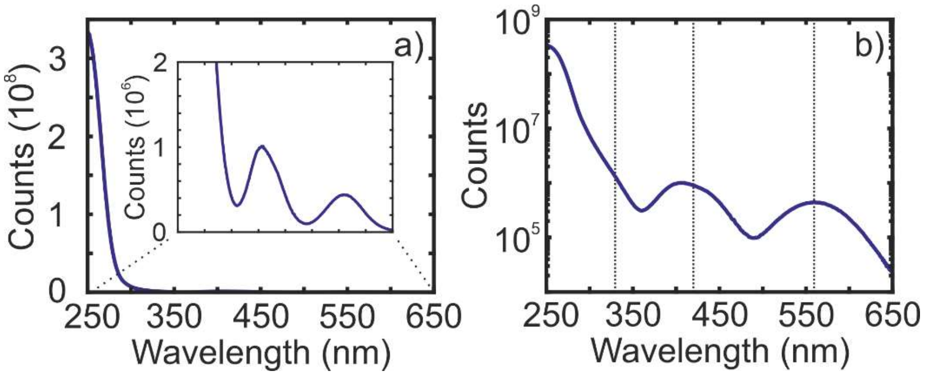

Figure S5. Photoluminescence excitation spectra in linear (a) and logarithmic (b) scale. Dotted curves in (b) represent the observed Cr^{3+} excitation lines: 330 nm, 420 nm and 560 nm.

Luminescence spectra under 260 nm excitation

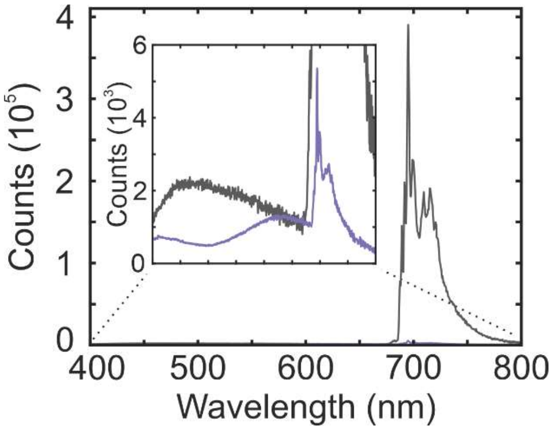

Figure S6. Photoluminescence spectra under 260 nm (grey line) and 330nm (purple line) excitation at 80 K. Inset shows the ZGO matrix emission centred at ~450 nm.

Calculated persistent luminescence for different times after excitation stops

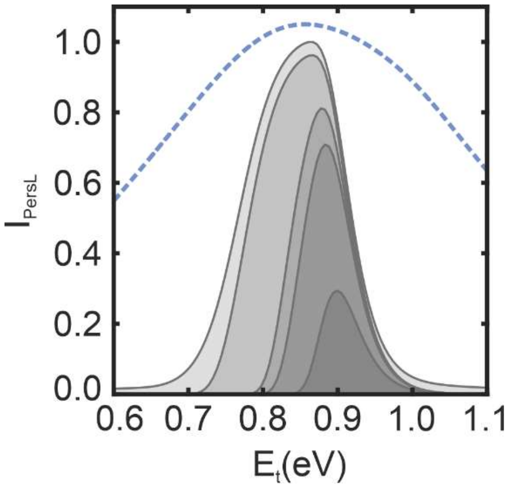

Figure S7. Zoom of Figure 4b of the main manuscript. Integrated persistent luminescence calculated for different times after excitation. From lighter to darker gray: 0 s, 1 s, 30 s, 1 min, 5 min. The full trap depth distribution is also plotted (dotted blue line) for visual reference.
