# Supplementary material for: Trap Depth Distribution Determines Afterglow Kinetics: A Local Model Applied to ZnGa2O4:Cr3+
Source: J Phys Chem Lett. 2024 Aug 29;15(35):9129–35. doi: 10.1021/acs.jpclett.4c01296 (PMC11382276; doi:10.1021/acs.jpclett.4c01296)
Supplement: Supplementary file 2 — jz4c01296_si_002.pdf [file jz4c01296_si_002.pdf]

jz-2024-012969.R1

Name: Peer Review Information for "Trap Depth Distribution Determines Afterglow Kinetics: A Local Model Applied to ZnGa₂O₄:Cr³⁺"

First Round of Reviewer Comments

Reviewer: 1

Comments to the Author

Requiring the Reviewer to write 1, 2, 3 sections is rather strange. These should be given in the manuscript. As written, it is difficult to read. It seems to be rushed and incomplete with no clear focus. It requires a major structured rewrite.

1. Define mtn on the right hand side of Eq 1.

2. The definition of second-order kinetics given - whereas second-order kinetics represents a non-local PersL mechanism in which free electron and hole recombination can occur – is unusual and does not refer to back transfer. Please explain.

3. Please include more description of “the density” of species, and its units.

4. It needs to be demonstrated that the TL intensity charging at low and room temperature is comparable.

5. It seems unlikely that the broad 630 nm emission is due to Cr3+ ions located in highly distorted lattice sites. In that case, why is it broad?

6. 6. The symbols in Eq 5 are not defined, particularly Gamma tot.

7. Please label Fig 2a as in text.

8. The Eq 3 should be charge conservative.

9. Why is Table 1 triexponential for 330 nm? What does that mean?

10. I do not understand the OSL effect as shown in the SI.

The quality of the figures is very poor and needs improvement.

Reviewer: 2

Comments to the Author

Romero et al. characterize persistent luminescence and thermoluminescence from $ZnGa_2O_4:Ce^{3+}$ nanoparticles. They present a model of persistent luminescence and thermoluminescence due to a trap state distribution. The model yields a good match with the experimental data. These phenomena due to long charge-carrier trapping are notoriously difficult to understand and the authors made a convincing case for their model. The analysis allows the authors to highlight possible improvements to persistent luminescence materials. The description of the model is well written (apart from some issues, mentioned below) and it could be implemented by other researchers working on this topic. This work will be of interest to them. I recommend that the work is suitable for J. Phys. Chem. Lett.

Nevertheless, I have a few questions/concerns:

(1) I am confused by the expression for p_1 in equation 4. The authors discuss at the end of the manuscript that retrapping can be important in certain scenarios. The assumption of a constant T_0 (equation 4) suppresses retrapping during thermoluminescence significantly. I do not understand the justification for this assumption and it could be influential.

(2) The manuscript does not explain how the distribution rho(E_t) is fitted. Are the values of rho(E_t^i) just N independent fit parameters? The smooth profile obtained (Fig. 3) suggests that the relative values of rho(E_t^i) are somewhat restricted. If the persistent luminescence is due to only a limited range of existing trap depths (Fig. 4b), how is the model able to determine the trap distribution outside this range?

(3) What does the gray line in Fig. 4a mean? Why is m_t^i limited to a maximum value of 10^0 in Fig. 4a, although $M = 10^{10}$?

(4) The authors discuss that the deepest traps would take a year to discharge. What does this mean for the charging process? Does it take equally long to reach a steady-state trap occupation? The experimental curve at the lowest power does not seem to reach a steady-state intensity level yet at 300 s.

(5) I recommend that in Fig. 4b the authors make a clearer distinction between the shades of gray of the distributions of discharging traps, and the lightest-gray shading of the full trap distribution. The full trap distribution is easily mistaken for the first spectrum in the series from lighter to darker gray.

Author's Response to Peer Review Comments:

Seville, May 28th 2024

Response letter to reviewers.

Response to Reviewer 1 (R1)

R1: *This paper may be publishable, but major revision is needed; I would like to be invited to review any future revision.*

Authors (A): We are pleased that the reviewer considers our manuscript publishable. Below we address all of the points raised for revision.

R1: *1. Define m_t on the right-hand side of Eq 1.*

A: We have included this definition in the new version of the manuscript. We thank the reviewer for pointing out this oversight.

R1: *2. The definition of second-order kinetics given - whereas second-order kinetics represents a non-local PersL mechanism in which free electron and hole recombination can occur – is unusual and does not refer to back transfer. Please explain.*

A: Light emission in semiconductors is typically modeled by two sets of rate equations that describe the time dependence of the electron density (n) in the conduction band (CB) and the hole density (h) in the valence band (VB). Since emission results from electron-hole recombination, the probability of a photon being emitted is proportional to the de product n·h, n^2 for undoped semiconductors. Thus, if only radiative recombination occurs, the rate equation for the electron density in the CB is

$$\frac{dn}{dt} = -kn^2 \tag{1}$$

where k is a constant. Eq. 1 then stablishes that the rate of n is proportional to n to the power of 2, which connects to second-order kinetics. The mechanism is then called non-local because charges (electrons and holes) move through the CB and the VB until they reach a recombination center. This allows the interaction

INSTITUTO DE CIENCIA DE MATERIALES DE SEVILLA Avd./ AMÉRICO VESPUCIO, 49 41092 SEVILLA Tel.: (34) 954 48 95 30

g.lozano@icsic.es

between distant traps and recombination centers, as it occurs in semiconductors, which makes the description of the mechanism *non-local*. In contrast, if a trap only interacts with its nearest recombination center, as it happens when Cr^{3+} ions are directly excited in $ZnGa_2O_4:Cr^{3+}$ [*Chem. Mater.* 2014, **26**, 1365-1373] or Eu^{2+} ions in $Sr_2MgSi_2O_7:Eu^{2+},Dy^{3+}$ [*Opt Mater Express* 2016, **6** (3), 844], the emission mechanism is called *local*. We have furthered discussed this relevant point in the revised version of our manuscript.

R1: *3. Please include more description of "the density" of species, and its units.*

A: The density of excited Cr^{3+} ions (m_e) and the density of charged traps (m_t) represent charges per unit volume. Thus, they have units of length to the power of -3, which is m^{-3} in the international system. However, since the rest of the parameters of our model are volume-independent (p_e, E_1, α, etc.), we decided to keep the densities unitless. In any case, the results are independent of the units used for the densities, as it only introduces a scaling factor. We have explained this in the revised version of the manuscript, following the reviewer's recommendation.

R1: *4. It needs to be demonstrated that the TL intensity charging at low and room temperature is comparable.*

A: Fig. R1 shows the thermoluminescence curves fitted in the manuscript before intensity normalization. It can be observed that both curves have similar counts at their maxima, regardless of charging occurring at low temperature (15K, blue line) or room temperature (300K, orange line). In view of the reviewer's comment, we have included this information in the Supporting Information of the revised manuscript.

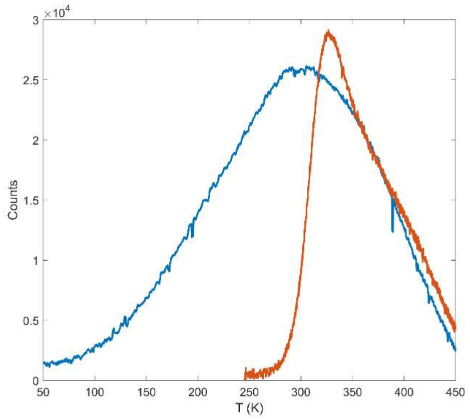

.

INSTITUTO DE CIENCIA DE MATERIALES DE SEVILLA
Avd./ AMÉRICO VESPUCIO, 49
41092 SEVILLA Tel.: (34) 954 48 95 30
g.lozano@icsic.es

Figure R1. New Figure S2. Thermoluminescence curves for two charging temperatures (blue line corresponds to charging at 15 K, while orange corresponds to charging at 295 K and then cooling down to 245 K before heating).

R1: *5. It seems unlikely that the broad 630 nm emission is due to Cr^{3+} ions located in highly distorted lattice sites. In that case, why is it broad?*

A: We agree with the reviewer that the origin of the broad band centered at 630 nm is debatable and further research is needed to unravel it. However, we are certain that it does not originate from Cr^{3+} $^2E \rightarrow ^4A_2$ transitions, which are inherently narrow. Furthermore, this band is only observed when the sample is measured at low temperature and does not appear in the PersL spectrum. Therefore, regardless of its origin, it is outside the scope of our model and does not affect the results of our analysis. We have commented on this point in the new version of the manuscript, following the reviewer's suggestion.

R1: *6. The symbols in Eq 5 are not defined, particularly Γ_{tot}.*

A: Γ_{tot} is the total decay rate. Following the reviewer's suggestion, in the revised version of our manuscript we have checked all the equations to ensure that each variable and coefficient is correctly defined.

R1: *7. Please label Fig 2a as in text.*

A: Following the reviewer's recommendation, we have changed the label of the vertical axis of panel c to be consistent with the text (see Fig. R2).

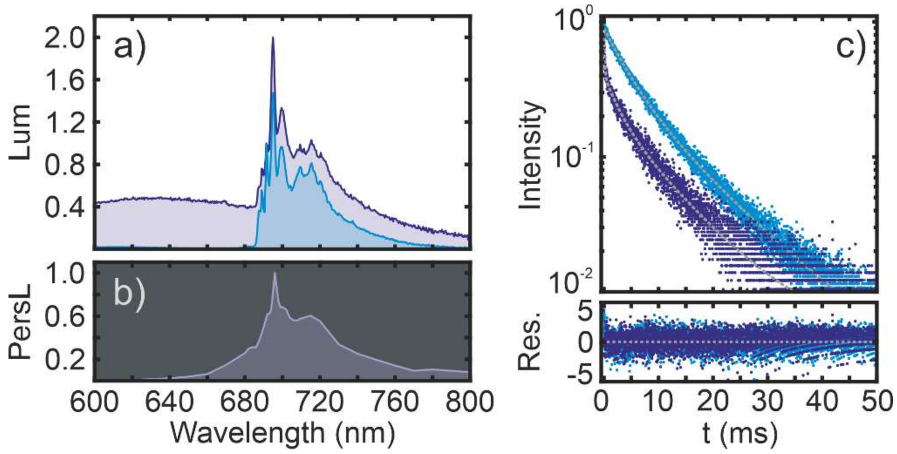

Figure R2. New Figure 2.

R1: *8. The Eq 3 should be charge conservative.*

.
INSTITUTO DE CIENCIA DE MATERIALES DE SEVILLA
Avd./ AMÉRICO VESPUCIO, 49
41092 SEVILLA Tel.: (34) 954 48 95 30
g.lozano@icsic.es

A: The referee is right. Eq. 3 in the original manuscript is indeed conservative. We did not choose the right words to emphasize that in this material, unlike the well-known $SrAl_2O_4:Eu^{2+},Dy^{3+}$ where charge trapping leads to a change in the oxidation state of Eu^{2+}, recombination centers remain optically active during charge trapping. We apologize for this misunderstanding. We have addressed this point in the new version of the manuscript and included a new schematic figure in the Supporting Information (see Figure R3).

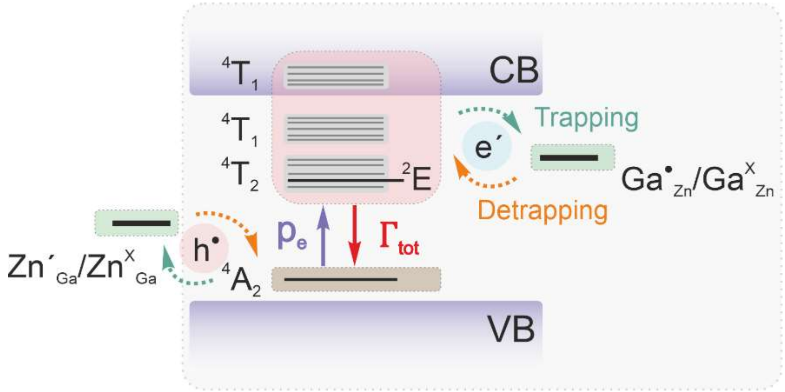

Figure R3. New Figure S1. Schematic of the complete trapping model with Cr^{3+} ions near antisite defects. The shaded areas correspond to those in Figure 1 of the main manuscript.

R1: *9. Why is Table 1 triexponential for 330 nm? What does that mean?*

A: We use a biexponential model to obtain the decay rate of the Cr^{3+} $^2E \rightarrow ^4A_2$ transitions involved in the afterglow. In fact, the two exponentials account for Cr^{3+} cations in slightly different crystalline environments, which provides a good fit to the time-dependent intensity measurements for 450 nm light excitation. For this photoexcitation condition, the PL and PersL spectra show only bands associated with Cr^{3+} $^2E \rightarrow ^4A_2$ transitions. However, to fit the experimental data under 330 nm light excitation, we must add an additional exponential to account for the fast contribution associated with the broad band centered at 630 nm that appears in the PL spectrum when it is measured at low temperature under UV excitation. Note that the average decay rate of the two exponentials associated with Cr^{3+} remains approximately constant regardless of the photoexcitation conditions. We have commented on this in the revised version of the manuscript.

R1: *10. I do not understand the OSL effect as shown in the SI.*

A: As PersL is the emission mechanism that originates from thermal detrapping at room temperature, OSL accounts for the emission from optical detrapping (OSD). To avoid any confusion, we would like to emphasize that OSL has demonstrated great potential for theranostics [*Theranostics* 2014, **4**(11), 1112-1122, doi:

.

INSTITUTO DE CIENCIA DE MATERIALES DE SEVILLA Avd./ AMÉRICO VESPUCIO, 49

41092 SEVILLA Tel.: (34) 954 48 95 30

g.lozano@icsic.es

10.7150/thno.9710]. However, an analysis of the stringent effect of OSD on the trapping capabilities of PersLNPs remains elusive. In particular, Fig. S4 shows time-dependent calculations of light emission assuming the fitting parameters shown in Table 2 of the original manuscript, but with α=0 to suppress optical detrapping. Our results indicate that OSD affects not only trap release, but also charging. Indeed, in the absence of optical detrapping, the charging curves show the same curvature regardless of the excitation power. Moreover, this effect causes charge detrapping to be faster under excitation, which simultaneously reduces the number of traps available under continuous excitation. Indeed, simulations with α=0 (dotted lines) show much higher afterglow than our measurements, with a strong dependence on the excitation power. As a result, we find that preventing optical detrapping during charging is key to increase the afterglow in PersL materials. We have further clarified this relevant point in the new version of the manuscript.

R1: *11. The quality of the figures is very poor and needs improvement.*

A: We have carefully revised all figures following the advice of the reviewer.

.

INSTITUTO DE CIENCIA DE MATERIALES DE SEVILLA Avd./ AMÉRICO VESPUCIO, 49

41092 SEVILLA Tel.: (34) 954 48 95 30

g.lozano@icsic.es

Response to Reviewer 2 (R2)

R2: *This paper is publishable subject to minor revisions noted. Further review is not needed. Romero et al. characterize persistent luminescence and thermoluminescence from* $ZnGa_2O_4:Cr^{3+}$ *nanoparticles. They present a model of persistent luminescence and thermoluminescence due to a trap state distribution. The model yields a good match with the experimental data. These phenomena due to long charge-carrier trapping are notoriously difficult to understand and the authors made a convincing case for their model. The analysis allows the authors to highlight possible improvements to persistent luminescence materials. The description of the model is well written (apart from some issues, mentioned below) and it could be implemented by other researchers working on this topic. This work will be of interest to them. I recommend that the work is suitable for J. Phys. Chem. Lett.*

A: We are glad the reviewer fully supports the publication of our manuscript in JPCL.

R2: *Nevertheless, I have a few questions/concerns.*

1. I am confused by the expression for p_1 *in equation 4. The authors discuss at the end of the manuscript that retrapping can be important in certain scenarios. The assumption of a constant* T_0 *(equation 4) suppresses retrapping during thermoluminescence significantly. I do not understand the justification for this assumption and it could be influential.*

A: The trapping rate p_1 is not temperature dependent in our model. Thus, retrapping can only affect TL measurements if p_1 is sufficiently high compared to Γ_{tot}, which is not the case for our material. We have clarified this point in the new version of the manuscript.

R2: *2. The manuscript does not explain how the distribution* $\rho(E_t)$ *is fitted. Are the values of* $\rho(E_t^i)$ *just N independent fit parameters? The smooth profile obtained (Fig. 3) suggests that the relative values of* $\rho(E_t^i)$ *are somewhat restricted.*

A: The reviewer is correct. The fitting of $\rho(E_t)$ was done by assuming that the trap distribution is a sum of several Gaussian distributions. In particular, the number of fitting parameters for $\rho(E_t)$ was set to 12 (4 gaussians defined by 3 parameters each: center, height and width). We have clarified this point following the advice of the reviewer.

.

INSTITUTO DE CIENCIA DE MATERIALES DE SEVILLA Avd./ AMÉRICO VESPUCIO, 49

41092 SEVILLA Tel.: (34) 954 48 95 30

g.lozano@icsic.es

R2: *If the persistent luminescence is due to only a limited range of existing trap depths (Fig. 4b), how is the model able to determine the trap distribution outside this range?*

A: PersL at a given temperature comes from a limited range of trap depths, as pointed out by the reviewer. For this reason, our analysis includes thermoluminescence measurements in order to infer information about the full range of trap depths. We have commented on this interesting subtlety in the revised version of the manuscript.

R2: *3. What does the gray line in Fig. 4a mean?*

A: The gray line in Figure 4a corresponds to the calculated PersL intensity 300s after the end of excitation. We have addressed this issue in the new version of the manuscript.

R2: *Why is m_t^i limited to a maximum value of 10^0 in Fig. 4a, although $M = 10^{10}$?*

A: The values of m_t^i shown in Figure 4a of the original manuscript are normalized for the sake of clarity. Before normalization, the actual values are on the order of M, i.e. 10^{10}. We have clarified this point in the revised version of the manuscript.

R2: *4. The authors discuss that the deepest traps would take a year to discharge. What does this mean for the charging process? Does it take equally long to reach a steady-state trap occupation? The experimental curve at the lowest power does not seem to reach a steady-state intensity level yet at 300 s.*

A: The time required to reach steady state is given by an equilibrium between all the different rates. Although deep traps are indeed associated with a low detrapping rate, p_2 is significantly smaller than p_1 and Γ_{tot}. To clarify this point, we can look at the solution of the following equation (see the Supporting Information for details):

$$\begin{pmatrix} m_e(t) \\ m_t(t) \end{pmatrix} = C_1 e^{\lambda_1 t} \boldsymbol{v_1} + C_2 e^{\lambda_2 t} \boldsymbol{v_2} \quad (2)$$

The time to reach the steady state is determined by $\lambda_{1,2}$, which is given by:

$$\lambda_{1,2} = -\frac{1}{2}\left(p_1 + p_2 + (1+\alpha)p_e + \Gamma_{tot} \pm \sqrt{(p_1 + p_2 + (\alpha - 1)p_e - \Gamma_{tot})^2 + 4p_1\Gamma_{tot}}\right) \quad (3)$$

For deep traps, p_2 tends to zero and $\lambda_{1,2}$ depends mainly on p_1, p_e, and Γ_{tot}. This indicates that the time required to reach equilibrium for charging deep traps is close to that for shallow traps. Finally, the fact that the experimental curve at the lowest power does not seem to reach a steady intensity at 300 s, is related to the

.

INSTITUTO DE CIENCIA DE MATERIALES DE SEVILLA

Avd./ AMÉRICO VESPUCIO, 49

41092 SEVILLA Tel.: (34) 954 48 95 30

g.lozano@icsic.es

dependence of $\lambda_{1,2}$ on p_e (see Figure R4). We have commented on this point in the new version of the manuscript and included Figure R4 in the Supporting Information.

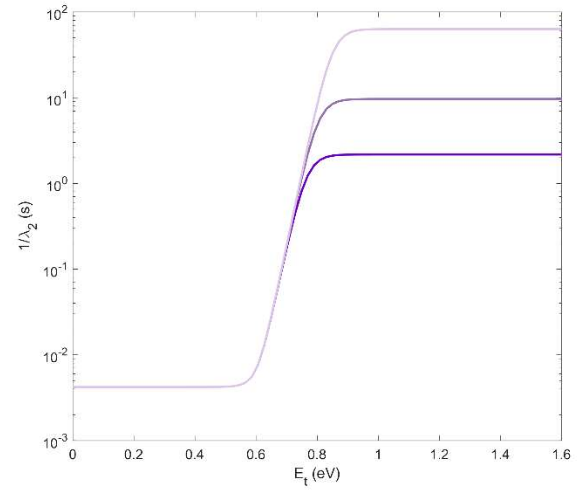

Figure R4. λ_2^{-1}as a function of trap depth E_t for the three values of the excitation intensity used in the main manuscript.

R2: *5. I recommend that in Fig. 4b the authors make a clearer distinction between the shades of gray of the distributions of discharging traps, and the lightest-gray shading of the full trap distribution. The full trap distribution is easily mistaken for the first spectrum in the series from lighter to darker gray.*

A: In the new version of the manuscript, we have changed this as suggested by the reviewer (see Figure R5). Also, a zoom in the range of 0.6 to 1.1 eV has been added in the Supporting Information (see Figure R6).

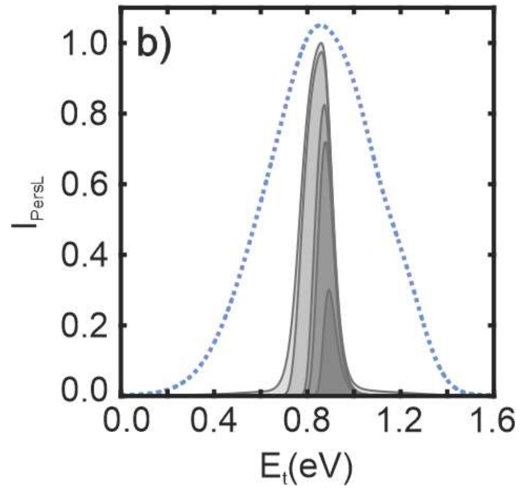

Figure R5. New Figure 4b.

INSTITUTO DE CIENCIA DE MATERIALES DE SEVILLA Avd./ AMÉRICO VESPUCIO, 49

41092 SEVILLA Tel.: (34) 954 48 95 30

g.lozano@icsic.es

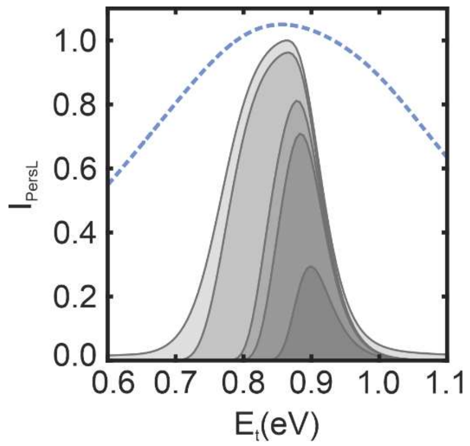

Figure R6. Zoom of Figure 4b of the main manuscript.

.

INSTITUTO DE CIENCIA DE MATERIALES DE SEVILLA Avd./ AMÉRICO VESPUCIO, 49

41092 SEVILLA Tel.: (34) 954 48 95 30

g.lozano@icsic.es

jz-2024-012969.R2

Name: Peer Review Information for "Trap Depth Distribution Determines Afterglow Kinetics: A Local Model Applied to ZnGa₂O₄:Cr³⁺"

Second Round of Reviewer Comments

Reviewer: 1

Comments to the Author

I hope that the authors do not mind if I raise a few more points.

I do find the terminology employed to be confusing to me and apologize for my lack of understanding – herein, local denotes 1st order kinetics and non-local (involving electron and hole recombination via CB) is 2nd order kinetics. In the review of Bos (Radiation Measurements 41 (2007) S45–S56) the latter process is labeled 1st order kinetics. Retrapping denotes 2nd order kinetics. I think my confusion arises from the use of the words trapping, retrapping and detrapping. Thus, could the authors please define these three words.

I understand that excitation of the activator site can occur by direct transfer from the trap (local transfer), by tunneling, or through thermal excitation of the trap electron into the CB. The authors write that since excitation is below the CB the process is 1st order. Certainly, the shape of the TL curves is representative of 1st order kinetics.

The broad emission band is said to have no effect upon the kinetics of the process. However, its inclusion in the measurements reverses the weighting of gamma 1 and gamma 2. How can this be?

The lifetimes in Figure 2 are measured at 695 nm. How would the values differ at 691.5 nm? The fitted parameters refer to 80 K. How can they be used to explain RT TL?

Author's Response to Peer Review Comments:

Seville, May 29th 2024

Dear editor,

Please find attached a new revised version of the manuscript "Trap Depth Distribution Determines Afterglow Kinetics: A Local Model Applied to $ZnGa_2O_4$:Cr^{3+}" (Research Article, No. jz-2024-012969.R1), by M. Romero et al. We are pleased that reviewers fully support the publication of our manuscript without further revision.

We have considered the final suggestions made by the reviewers and by the editorial office and have modified the manuscript accordingly. To make the changes easy to follow, they are highlighted in yellow in the new version and explained in the response letter. We believe that the overall quality of the manuscript has improved as a result of the review process, and we are grateful for this. We hope that the new version of our manuscript is now suitable for publication in your journal.

Sincerely,

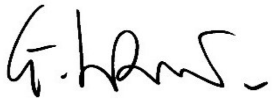

Dr. Gabriel Lozano
Tenured Scientist of the Spanish National Research Council at the Institute of Materials Science of Seville

INSTITUTO DE CIENCIA DE MATERIALES DE SEVILLA.

Avd./ AMÉRICO VESPUCIO, 49

41092 SEVILLA Tel.:
(34) 954 48 95 30

g.lozano@icsic.es
